# Synthesis and Biological Activity Evaluations of Green ZnO-Decorated Acid-Activated Bentonite-Mediated Curcumin Extract (ZnO@CU/BE) as Antioxidant and Antidiabetic Agents

**DOI:** 10.3390/jfb14040198

**Published:** 2023-04-04

**Authors:** Hassan Ahmed Rudayni, Marwa H. Shemy, Malak Aladwani, Lina M. Alneghery, Gasem M. Abu-Taweel, Ahmed A. Allam, Mostafa R. Abukhadra, Stefano Bellucci

**Affiliations:** 1 Department of Biology, College of Science, Imam Muhammad bin Saud Islamic University, Riyadh 11623, Saudi Arabia; 2 Chemistry Department, Faculty of Science, Beni-Suef University, Beni-Suef 65211, Egypt; 3 Materials Technologies and Their Applications Lab, Geology Department, Faculty of Science, Beni-Suef University, Beni-Suef 65214, Egypt; 4 Department of Biology, College of Science, Jazan University, P.O. Box 2079, Jazan 45142, Saudi Arabia; 5 Zoology Department, Faculty of Science, Beni-Suef University, Beni-Suef 62514, Egypt; 6 Geology Department, Faculty of Science, Beni-Suef University, Beni-Suef 65214, Egypt; 7 INFN-Laboratori Nazionali di Frascati, Via. E. Fermi 54, 00044 Frascati, Italy

**Keywords:** bentonite, ZnO, curcumin, composite, antioxidant, antidiabetes

## Abstract

Green ZnO-decorated acid-activated bentonite-mediated curcumin extract (ZnO@CU/BE) was prepared as a multifunctional antioxidant and antidiabetic agent based on the extract of curcumin, which was used as a reducing and capping reagent. ZnO@CU/BE showed notably enhanced antioxidant properties against nitric oxide (88.6 ± 1.58%), 1,1-diphenyl-2-picrylhydrazil (90.2 ± 1.76%), 2,2′-azino-bis(3-ethylbenzothiazoline-6-sulphonic acid (87.3 ± 1.61%), and superoxide (39.5 ± 1.12%) radicals. These percentages are higher than the reported values of ascorbic acid as a standard and the integrated components of the structure (CU, BE/CU, and ZnO). This signifies the impact of the bentonite substrate on enhancing the solubility, stability, dispersion, and release rate of the intercalated curcumin-based phytochemicals, in addition to enhancing the exposure interface of ZnO nanoparticles. Therefore, effective antidiabetic properties were observed, with significant inhibition effects on porcine pancreatic α-amylase (76.8 ± 1.87%), murine pancreatic α-amylase (56.5 ± 1.67%), pancreatic α-glucosidase (96.5 ± 1.07%), murine intestinal α-glucosidase (92.5 ± 1.10%), and amyloglucosidase (93.7 ± 1.55%) enzymes. These values are higher than those determined using commercial miglitol and are close to the values measured using acarbose. Hence, the structure can be applied as an antioxidant and antidiabetic agent.

## 1. Introduction

Diabetes is a widely distributed clinical pancreatic syndrome whose symptoms eventually increase in subsequent periods and is predicted to be the seventh leading cause of mortality. According to the World Health Organization (WHO), 366 million individuals will be diagnosed by 2030 [1,2,3]. Diabetes is classified into type 1 diabetes mellitus (T1DM)) and type 2 diabetes mellitus (T2DM), which will represent 90% of patients with diabetes by 2030 [1,4]. T2DM is a severe metabolic disorder associated with postprandial hyperglycemia (significant levels of glucose) and abnormal elevation of the levels of released free radicals (reactive oxygen) [5,6,7]. The hyperglycemia and the increase in blood glucose concentrations are affected mainly by the levels of the generated oxidative enzymes such as α-amylase, α-glucosidase, and amyloglucosidase enzymes. Such enzymes accelerate strongly the breakdown reactions of complex sugars into glucose and its absorption rate [5,7]. Chronic hyperglycemia causes a significant increase in the production of advanced glycation products (AGEs) that significantly affect the pathogenesis associated with diabetic complications [8,9]. The release of the reactive oxygen species results in the development of pathophysiological conditions involving the weakening of antioxidant defense mechanisms [7,10,11]. Additionally, oxidative stress is an essential factor involved in inducing insulin resistance, lipid peroxidation, and the destruction of cellular organelles and blood vessels [8,9,12].

Several chemical antidiabetic drugs (biguanides, miglitol, sulfonylureas, acarbose, thiazolidinedione, and voglibose) have been used effectively to reduce hyperglycemia and oxidizing radicals [7,9]. However, they do not exhibit adequate long-term effects and are associated with adverse health issues such as abdominal distention, diarrhea, hepatotoxicity, meteorism, and severe hypoglycemia [13,14]. Therefore, several studies have been conducted to design multifunctional structures with significant antioxidant and antidiabetic properties, strong preventive effects on the generation of oxidative stress, and the ability to manage T2DM [7,15]. Recently, metal and metal oxide nanostructures have been extensively investigated for this purpose owing to their reported biological activities, surface area, and physicochemical properties [15,16,17]. Moreover, they exhibit significant therapeutic and theranostic potentiality in addition to their remarkable safe effects on healthy cells and tissues [16,18]. Numerous edible plant species have been categorized as antioxidant and antidiabetic-safe products [9,15,19]. This is related to their phytochemicals constituents (polyphenols, tannins, glucosinolates, terpenes, steroids, and carotenoids), which can reduce the level of sugar in the blood as well as the commonly reported risks of T2DM and its complications [10,20,21].

Zinc oxide and its nanomaterials have been reported as promising, safe, biocompatible, and non-toxic antidiabetic and antioxidant agents that exhibit notable preventive effects on the generation of free radicals [17,22,23]. Zinc is an essential and vital element in human organs and plays a significant role in the synthesis of nucleic acids and proteins [16,23,24]. Moreover, it is widely used in biomedical applications such as tissue engineering and drug delivery and in the development of antioxidant, antibacterial, anticancer, and hypoglycemic agents [16,25]. Recent studies have demonstrated the significant effects of synthesis methods, mechanical stability, morphology, hybridization, and surface modification of zinc oxide-based materials on their biomedical properties [16,17,26]. Moreover, the formation of complexes between zinc-based structures and common phytochemical compounds or their synthesis using plant-associated methods confers biocompatibility, genotoxicity, and antioxidant properties to the compounds [22,23,27,28]. Therefore, the green synthesis of zinc oxide based on extracts of plants, which are enriched with well-known antioxidant phytochemicals, such as *turmeric rhizome,* may lead to compounds with significant antidiabetic properties. Green synthesis methods have been widely recommended over the last decade as ecofriendly, cost-effective, simple, nontoxic, biosafe, and highly productive synthesis methods for nonagglomerated nanoparticles [17,29]. Moreover, the resulting particles are commonly coated with capping films of valuable phytochemicals, such as alkaloids, phenolic compounds, amino acids, and proteins [17].

Curcumin (CU) is a natural polyphenolic compound of low molecular weight extracted from *turmeric rhizome* (*Curcuma longa*). Curcumin is a potent natural antibacterial, antioxidant, antitumor, anti-inflammatory, and anticancer agent [27,30]. However, medical applications of curcumin are limited because of its low chemical stability, low water solubility, poor bioavailability, and fast metabolism properties [17,31,32]. Recent studies have demonstrated significant enhancement upon incorporation of curcumin into suitable carriers, as well as its conjugation with metal and metal oxides on its solubility and bioactivity as an antioxidant, anti-inflammatory, and antimicrobial agents [33,34,35]. The application of natural bentonite as a substrate for zinc oxide and zinc/curcumin complex, as well as a host for curcumin, will result in an innovative and enhanced multifunctional antioxidant and antidiabetic agent [24]. Bentonite is a common natural biocompatible clay mineral widely used in numerous pharmaceutical and medical applications and in drug delivery systems [36,37,38] owing to its notable natural availability, high surface area, ion exchange capacity, adsorption and absorption properties, surface reactivity, and nontoxicity [36,39].

Therefore, the presented study aimed to synthesize ZnO-decorated bentonite-mediated curcumin extract (ZnO@CU/BE) by facile organic intercalation of the curcumin extract followed by green decoration of ZnO using the same curcumin extract as the reducing and capping reagent. The synesthetic composite was characterized as enhanced multifunctional antioxidant and antidiabetic agents with several species of bioactive compounds (curcumin-based phytochemicals, zinc oxide-capped curcumin, and zinc/curcumin complexes). 

## 2. Materials and Methods

### 2.1. Materials and Chemicals

The raw bentonite sample used during the preparation of the structure was obtained from a bentonite quarry in Western Desert, Egypt with 54.82% (SiO_2_), 17.56% (Al_2_O_3_), 9.5% (Fe_2_O_3_), 2.6% (Na_2_O), 2.5% (MgO), 2.4% (CaO), 1.45% (TiO_2_), and 9.2% (LOI)). The acid activation step of bentonite occurred using sulfuric acid (99% purity; Sigma-Aldrich, Cairo city, Egypt). Highly pure curcumin (>94.99; Sigma-Aldrich) and analytical-grade absolute ethanol were used to obtain the green reducing extract and curcumin solution. Zinc nitrate hexahydrate (Zn (NO_3_)_2_.6H_2_O) powder (98%; Sigma-Aldrich) salt is the incorporated precursor during the green support of ZnO nanoparticles into the bentonite carrier. α-amylase (EC Number: 232-565-6; CAS Number: 0009000902; Enzyme Commission number: 3.2.1.1 (BRENDA, IUBMB)), α-glucosidase (EC Number: 232-604-7; CAS Number: 9001-42-7; Enzyme Commission number: 3.2.1.20 (BRENDA, IUBMB)), amyloglucosidase (EC Number: 232-877-2; CAS Number: 9032-08-0; Enzyme Commission number: 3.2.1.3 (BRENDA, IUBMB)), Miglitol drug (C_8_H_17_NO_5_), acarbose drug (≥95%; C_25_H_43_NO_18_), para-nitrophenyl α-glucopyranoside (pNPG), glacial acetic acid, starch, ethylene diamine tetra acetic acid (EDTA), L-ascorbic acid, potassium persulfate, sodium nitroprusside, sulphanilic acid, nitroblue tetrazolium (NBT), 1,1-diphenyl-2-picrylhydrazil (DPPH), 2,2′-azino-bis(3-ethylbenzothiazoline-6-sulphonic acid (ABTS), *N*-(1-Naphthyl) ethylenediamine dihydrochloride, and phosphate buffer were delivered from Sigma-Aldrich, Egypt and were used in the antioxidant and antidiabetic tests. 

### 2.2. Synthesis of ZnO Decorated Acid Activated Benetonite Mediated Curcumin Extract (ZnO@CU/BE)

#### 2.2.1. Acid Activation of Bentonite

The acid activation step of bentonite was performed to remove metallic and carbonate impurities. The activation processes were accomplished using 100 mL of diluted sulfuric acid (20%) in the presence of 10 g of raw bentonite. Homogenization between the bentonite fractions and acid was performed for 12 h at 100 °C using a magnetic stirrer at an adjustable speed of 650 rpm. The activated bentonite was then separated from the acid solution by filtration using whatman filter paper, neutralized by washing with distilled water, and dried at room temperature (40.3 °C) for 48 h. Subsequently, the sample was maintained for further characterization and synthesis. 

#### 2.2.2. Synthesis of Curcumin Intercalated Bentonite (CU/BE)

**Figure 1:**
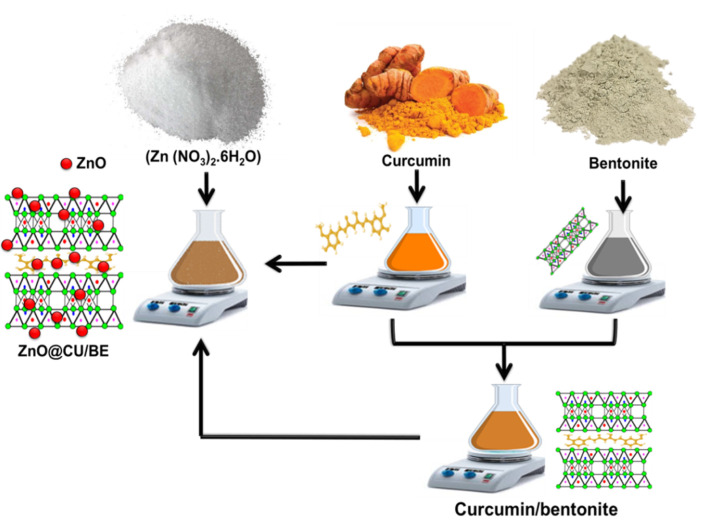
Schematic diagram for the synthesis procedures of curcumin intercalated bentonite (50 mL bentonite suspension ((4.4 g) + 50 mL water) + 50 mL curcumin extract (3.7 g of curcumin + 100 mL of ethanol (60%)) and ZnO@CU/BE composite (100 mL bentonite/zinc nitrate mixture (4.4 g bentonite + 2.2 g of Zn(NO_3_)_2_.6H_2_O + 100 mL water) + 100 mL curcumin extract (3.7 g of curcumin + 100 mL of ethanol (60%)).

Curcumin-intercalated bentonite was prepared to support the main phytochemicals within the layered silicate host of bentonite. First, approximately 3.7 g of curcumin was dispersed in 100 mL of ethanol (60%) for 15 min using a magnetic stirrer at 1500 rpm and gentle temperature at 50 °C. This mixing step by the magnetic stirrer was followed by an additional homogenization step involving sonication of the curcumin/ethanol mixture for approximately 20 min at a power of 240 W. The obtained curcumin extract (50 mL) was added to a previously prepared colloid solution of bentonite fractions (4.4 g of bentonite in 50 mL of distilled water). The bentonite/curcumin extract mixture was homogenized for 12 h with stirring at 1500 rpm. The mixture was then transferred to a sonication bath for an additional 5 h to confirm the successful intercalation of the bentonite layers with the phytochemicals of the curcumin extract. Finally, the obtained solid product was extracted from the residual solution, washed several times, and dried for 12 h at 50 °C (Figure 1).

#### 2.2.3. Synthesis of ZnO@ Benetonite Mediated Curcumin Extract (ZnO@CU/BE)

The green decoration of bentonite with ZnO nanoparticles involved the preparation of curcumin green extract as the applied reducing and capping reagent according to the procedures reported in Section 2.2.2. As a parallel step, 4.4 g of bentonite was dispersed within an aqueous solution of zinc nitrate (100 mL; 2.2 g of Zn (NO_3_)_2_.6H_2_O) with stirring for 10 h at 650 rpm. The solution was then subjected to sonication waves (250 W) for additional 2 h. The sonication step was followed by the addition of the curcumin extract (100 mL) to the prepared bentonite/zinc nitrate mixture with high-speed stirring until a dark red precipitate was detected. The stirring process was continued for 24 h at five intervals of 20 min of sonication to confirm the homogenous distribution of the formed zinc oxide or zinc oxide/curcumin complexes throughout the bentonite host. The product was extracted after this step by filtration, washed carefully with distilled water for several times, dried overnight at 50 °C, labeled as ZnO@CU/BE, and stored in glass containers at 25 °C for further characterization and testing (Figure 1).

### 2.3. Characterization Methods

The crystalline properties and structural effects of the compounds obtained after the modification steps were determined based on the X-ray diffraction patterns. The patterns were obtained using a PANalytical-Empyrean X-ray diffractometer with a measuring range of 5–80°. The functional chemical groups during the synthesis procedures were illustrated based on the FT-IR spectrum obtained using a Fourier-transform infrared spectrometer (FTIR−8400S; Shimadzu) with a measuring range of 400 cm^−1^ to 4000 cm^−1^. The morphological changes in terms of different modification steps were examined based on SEM images obtained using a scanning electron microscope (Gemini, Zeiss-Ultra 55) and HRTEM images obtained using a transmission electron microscope (JEOL-JEM2100). The textural properties in terms of the specific surface area and porosity were described based on the N_2_ adsorption/desorption isotherm curves determined using a surface area analyzer (Beckman Coulter SA3100).

### 2.4. Antioxidant Activities

#### 2.4.1. Nitric Oxide Radical Scavenging Assay

The scavenging of nitric oxide by the prepared structures was assessed based on the procedure reported by Kitture et al. [40]. The prepared solutions of the synthetic structures (20 μL; 100 μg/mL) were mixed with 2 mL of sodium nitroprusside (10 mM) in saline phosphate buffer solution (500 μL, pH 7.4). The mixed solutions were incubated for 150 min at 25 °C. By the end of the incubation period, 500 μL of the obtained mixture is taken and mixed with sulfanilic acid (1 mL), which was prepared by diluting 33% of it with 20% glacial acetic acid before incubation again for an additional 5 min. Naphthyl ethylenediamine dihydrochloride (1 mL; 0.1% *w*/*v*) was then added to the incubated mixture, which was re-incubated again for 30 min. By the end of the incubation process, the scavenging percentages of nitric oxide were calculated according to Equation (1) after determining the absorbance at 540 nm.

(1) $$Scavenging\;\left(\%\right)=\frac{A540_{Control}-A540_{Test}}{A540_{Control}}\times 100$$

#### 2.4.2. DPPH Radical Scavenging Assay

The efficiency of the synthetic structures as scavengers of DPPH radicals was assessed based on the method reported by Robkhob et al. [7]. The synthetic structures (20 μL; 100 μg/mL) were mixed homogenously in 96-well plates with methanolic solution (80 μL) composed of DPPH (100 μM). After the mixing step, the system was incubated in the dark for 20 min, and the changes in absorbance after the incubation period were determined using a microplate reader at an estimated wavelength of 517 nm. The measured values were used in the direct calculation of the scavenging percentages according to Equation (2).

(2) $$Scavenging\;\left(\%\right)=\frac{A517_{Control}-A517_{Test}}{A517_{Control}}\times 100$$

#### 2.4.3. ABTS Radical Scavenging Assay

The scavenging properties of the prepared materials were evaluated based on the procedures presented by Dappula et al. [41]. A stock solution of ABTS (7 mm) was prepared by dissolving 44 mg of ABTS in 10 mL of deionized water, which was mixed with another solution of potassium persulfate (3 μL) to generate ABTS radicals (ABTS^●+^) by incubating the system for 18 h in the dark at 25 °C. The generation of the ABTS^●+^ was followed by the addition of methanol at a ratio of 1:29 to obtain fresh ABTS^●+^. The prepared structures (10 μL; 100 μg/mL) were then mixed with the ABTS solution (290 μL) for 30 min. By determining the absorbance of the solutions in the absence (control) and presence of the structure (test) at 734 nm, the scavenging percentages of the ATBS radicals were calculated according to Equation (3).

(3) $$Scavenging\;\left(\%\right)=\frac{A734_{Control}-A734_{Test}}{A734_{Control}}\times 100$$

#### 2.4.4. Superoxide Radical Scavenging Assay

The scavenging efficiency of superoxide radicals released by the synthetic structures was assessed according to the procedure reported by Robkhob et al. [7]. The scavenging process involved mixing the prepared structures (100 μL; 100 μg/mL) with a riboflavin solution (100 μL; 20 μg), EDTA solution (200 μL; 12 mM), ethanol (0.1 mM; 200 μL), and NBT (100 μL; 0.1 mg). The obtained mixture was diluted with phosphate buffer (3 mL, 50 mM) and exposed to an illumination source for 5 min. By determining the absorbance of the solutions in the absence (control) and presence of the structure (test) at 540 nm, the scavenging percentages of the superoxide radicals were calculated according to Equation (4).

(4) $$Scavenging\;\left(\%\right)=\frac{A540_{Control}-A540_{Test}}{A540_{Control}}\times 100$$

### 2.5. Antidiabetic Studies

#### 2.5.1. Porcine Pancreatic α-Amylase Inhibition Assay

The antidiabetic properties of the synthetic structure were assessed based on the α-amylase inhibition assay using the previously reported chromogenic 3,5-dinitrosalicylic acid (DNSA) method [7]. The synthetic products (500 μL; 100 μg/mL) were incubated with porcine pancreatic α-amylase (100 μL; 50 μg/mL) for 10 min at 37 °C and followed by the careful addition of starch substrate (1.5 mL; 1%). The absorbance of α-amylase in the presence (test) and absence (control) of the synthetic products was measured at 540 nm, and the obtained results were used to calculate their inhabitation percentage according to Equation (5).

(5) $$Inhibition\;\left(\%\right)=\frac{A540_{Control}-A540_{Test}}{A540_{Control}}\times 100$$

#### 2.5.2. Crude Murine Pancreatic α-Amylase Inhibition Assay

The effect of the synthetic products on the enzymes of the crude murine model was assessed to confirm their potential as antidiabetic agents. The pancreas of a Swiss male mouse (ten-week-old) was used as the source of the studied enzymes, which was subjected to starvation for 12 h. Subsequently, it was homogenized and excised in saline phosphate buffer supplemented with protease inhibitors. This was followed by centrifugation at 10,000 rpm for 15 min to isolate cell-free supernatant. The isolated cells were diluted to obtain an absorbance of 0.4 at 280 nm and used as the tested source of crude enzyme. Crude murine pancreatic amylase was inhibited according to the steps described in Section 2.5.1.

#### 2.5.3. α-Glucosidase Inhibition Assay

The inhibitory effect of the synthetic products on α-glucosidase was investigated according to the methodology reported by Sanap et al. [42] The synthetic products (200 μL; 100 μg/mL) were mixed with 100 μL of the tested α-glucosidase (100 μL; 0.1 unit/mL), followed by incubation for 60 min at 37 °C. Then, 10 μL of p-nitrophenyl-α-d-glucopyranoside (pNPG) was added to the incubated mixture, which was incubated again for an additional 10 min at the same temperature. Subsequently, 2 mL of Na_2_CO_3_ (0.1 M) was added to the system to stop the reaction. The absorbance of nitrophenol diffused from pNPG was determined using a microplate reader at a wavelength of 420 nm, and the values were used to estimate the inhibition percentages according to Equation (6).

(6) $$Inhibition\;\left(\%\right)=\frac{A420_{Control}-A420_{Test}}{A420_{Control}}\times 100$$

#### 2.5.4. Crude Murine Intestinal α-Glucosidase Inhibition Assay

Swiss male mice were used as the source of crude intestinal α-glucosidase used during the test, considering the preparation procedures reported in the Section 2.5.2. Moreover, the inhibitory potential of the prepared products on crude murine intestinal α-glucosidase was studied according to the steps reported in Section 2.5.3 in the presence of a p-nitrophenyl-α-D-glucopyranoside substrate. 

#### 2.5.5. Amyloglucosidase Inhibition Assay

The inhibitory properties of the synthetic structures against amyloglucosidase were estimated according to Lawande et al. [43]. The prepared materials (100 μL; 100 μg/mL) were mixed with amyloglucosidase (100 μL; 0.1 unit/mL) and incubated at 37 °C for 10 min in the presence of starch substrate (1%). The absorbance of amyloglucosidase in the presence (test) and absence (control) of the synthetic products was measured at 540 nm, and the obtained results were used to calculate their inhabitation percentages according to Equation (5).

### 2.6. Statistical Analysis

The presented values in the study represent the mean values ± the standard error of the mean (S.E.M.; n = 3), and the significance of the statistical evaluation was assessed based on the analysis results of variance (ANOVA) as well as paired tests considering the value of * p < 0.05.

## 3. Result and Discussion

### 3.1. Characterization of the Synthetic Structure

#### 3.1.1. Structural Properties

**Figure 2:**
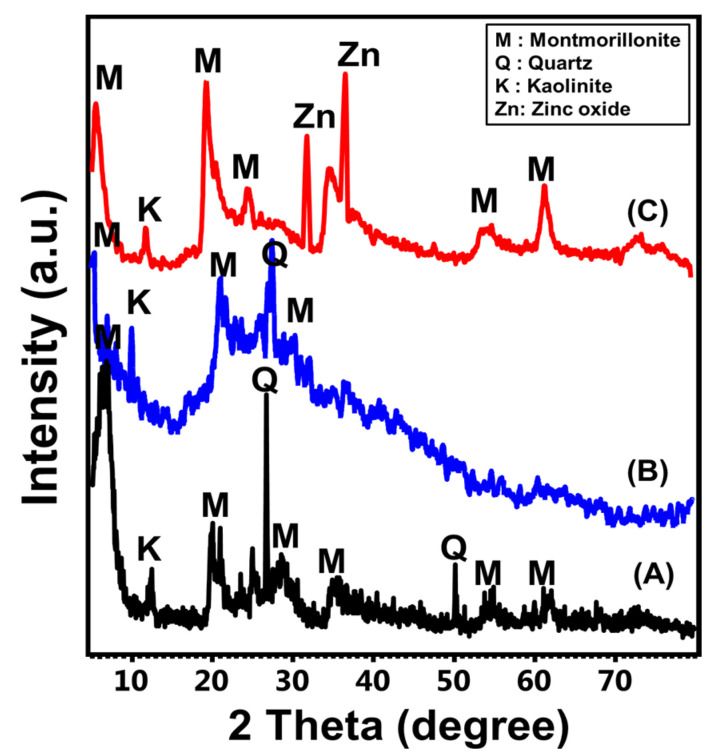
XRD patterns of raw bentonite (A), acid activated bentonite (B), the synthetic ZnO@CU/BE structure (C).

The crystalline and structural properties of raw bentonite, activated samples, and synthetic ZnO@CU/BE were assessed based on their XRD patterns. The resultant pattern of the raw sample shows the common diffraction peaks of bentonite. Montmorillonite was identified as the dominant and essential phase of clay minerals with peaks at 5.78° (001) and 6.95° (002), in addition to other peaks at 19.85°, 21.54°, 26.68°, and 28.56° (Ref. card No: 000-003-0010)) (Figure 2 (A)). In addition, the recognized pattern demonstrated the existence of clay (kaolinite) and non-clay (quartz) impurities, which are common in natural bentonite (Figure 2 (A)). The detected montmorillonite phase exhibited an average crystallite size of 12.9 nm and basal spacing of 12.71 Å. After the acid activation step, the resulting pattern demonstrated the apparent effects of sulfuric acid on the sample and structure of the montmorillonite phase (Figure 2 (B)). A remarkable decline was noted in the previously identified peaks of montmorillonite as well as a deviation in the position of the main peaks to lower angles (2Theta = 5.05° and 6.6°), which has been documented widely in acid-activated bentonite (Figure 2 (B)) [44,45,46]. Such changes in the XRD diffraction peaks validate the partial deformation impacts of the acid activation steps on the structure of bentonite, which might be associated with an enhancement in the surface reactivity and textural properties. 

Regarding the obtained pattern of the prepared ZnO@CU/BE, the recognized peaks revealed significant changes in the structure of bentonite as well as the identification of different crystalline phases (Figure 2 (C)). First, the obtained pattern reflected significant enhancement in intensifying some of the common montmorillonite peaks but at deviated positions (6.48°, 20.12°, and 25.35°) as well as the basal spacing value (13.61 Å) (Figure 2 (C)). The deviation in the positions of the peaks and the increment in the basal spacing validate the significant effect of successfully intercalated curcumin-related phytochemicals between the montmorillonite layers. The absence of peaks related to the curcumin structure suggested molecular-level dispersion as well as disordered crystalline properties of its structure as intercalated organic components within the montmorillonite layers [47]. Moreover, new peaks were detected at sharp intensities, signifying significant decoration of the curcumin/bentonite structure with green ZnO nanoparticles of the wurtzite structure (Figure 2 (C)). Good diffraction peaks of ZnO were observed at 31.65° (100) and 36.4° (101), in addition to other peaks that might overlap with some montmorillonite peaks, especially around 34° and 56° (Figure 2 (C)) (JCPDS no. 79-2205; JCPDS no. 65-3411).

#### 3.1.2. Morphological and Textural Properties

**Figure 3:**
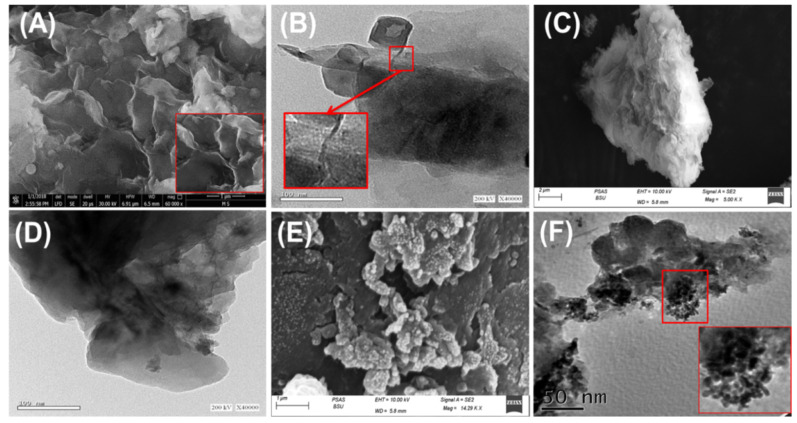
SEM image of raw bentonite (**A**) [48], HRTEM image of raw bentonite (**B**) [49], SEM images of synthetic CU/BE composite (**C**), HRTEM image of the synthetic CU/BE composite (**D**), SEM image of the synthetic ZnO@CU/BE compoaite (**E**), and HRTEM image of the synthetic ZnO@CU/BE composite (**F**).

The morphological features were significantly affected by the different modification and hybridization processes. The changes in the surface, as well as the internal features of bentonite during the different functionalization steps, were observed based on the SEM and HRTEM images (Figure 3). The raw bentonite appeared in the SEM images as aggregated clusters of compacted and stacked flake grains, which are common features of clay minerals. The high-magnification SEM images revealed the characteristic cornflake morphology of the montmorillonite grains with observable flexing and curvature of their platelets, forming notable lenticular secondary pores (Figure 3A). The studied HRTEM images of BE particles reflected their common internal structure, as they exhibited observable multilayered properties with a known lattice structure, which is a characteristic feature of the montmorillonite phyllosilicate structure (Figure 3B). The intercalation of the bentonite sheets with the extracted phytochemicals of curcumin exhibited considerable exfoliation effects, as the structural clay layers appeared to be displaced from each other in the SEM images (Figure 3C). The bentonite fractions appeared as swollen particles compared with the reported compacted aggregates of the raw sample (Figure 3C). This is in agreement with the results of HRTEM, as the bentonite grains appeared to be hybridized by polymeric films of nearly fibrous forms and rendered a darker gray tone to the grains than that of the commonly detected bentonite grains (Figure 3D). After the green decoration step of curcumin intercalated bentonite (CU/BE) particles with the ZnO nanoparticles, the SEM images reflected the remarkable distribution of the formed ZnO nanoparticles on their surfaces with irregular to spherical forms (Figure 3E). This was also confirmed by HRTEM images, which indicated the presence of metal oxide nanoparticles (ZnO) as inclusions between the intercalated bentonite layers and its surface (Figure 3F).

Previously reported morphological changes were associated with significant textural changes in terms of surface area and porosity properties. The surface area was enhanced notably from 91 m^2^/g for raw bentonite (BE) to 98.7 m^2^/g, 106.3 m^2^/g, and 118.4 m^2^/g for the prepared acid-activated BE, CU/BE, and ZnO@CU/BE, respectively. The enhancement in the surface area signifies the impact of the acid activation step on the surface area, as well as the effect of the intercalation process of the bentonite layers with extracts of curcumin in expanding the basal spacing, which is associated with an enhancement in the surface area. In addition, the presence of ZnO as decorated nanograins on the surface of the structure imparted an irregular typography to the surface, which induces surface area.

#### 3.1.3. Chemical Properties

**Figure 4:**
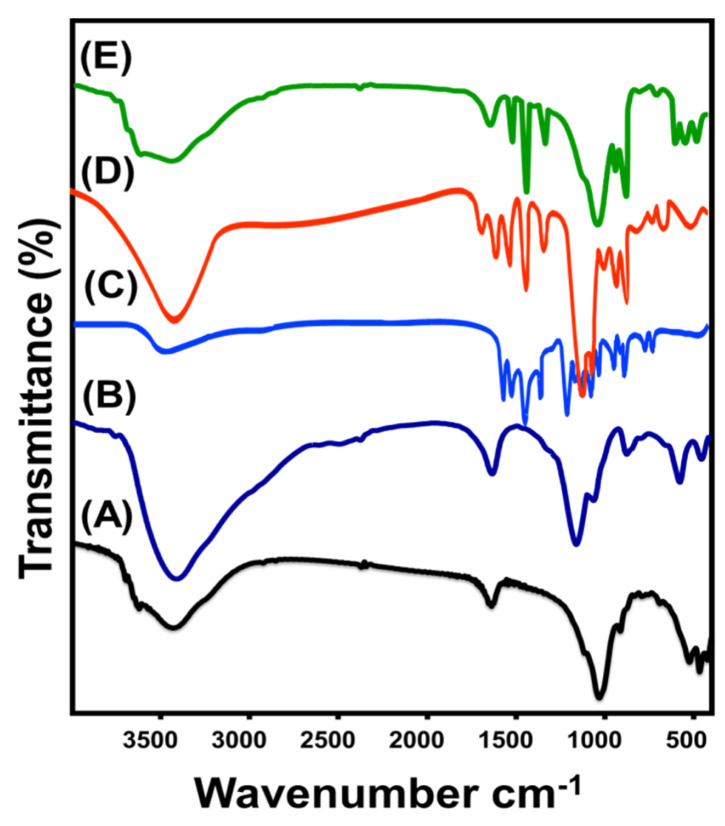
FT–IR spectrum of raw bentonite (A), acid activated bentonite bentonite (B), curcumin powder (C), synthetic CU/BE composite (D), and synthetic ZnO@CU/BE composite (E).

The modification effects on the chemical properties of the obtained structures as well as the dominant chemical groups were determined based on the FT–IR spectra of the prepared materials (Figure 4). The spectrum of the bentonite precursor clearly displayed the essential bands of structural –OH groups (3400 cm^−1^) that coordinate to the octahedral cations (Al_2_OH, AlMg(OH), and AlFe^3+^(OH)) and the characteristic interlayer molecules of the montmorillonite structure (1640 cm^−1^) (Figure 4 (A)). Moreover, the Si-O and Al-O groups were clearly signified by the observed bands at approximately 1000 cm^−1^ and 918 cm^−1^, respectively. The recognized bands within the determination area from 400 cm^−1^ to 1000 cm^−1^ corresponded to the bending vibrations of Si–O–Al (520 cm^−1^), Si-O-Si (466 cm^−1^), Si–O–Mg, and Mg–Fe–OH (Figure 4 (A)) [49,50]. The spectrum of bentonite after the acid activation step demonstrated no remarkable changes in the identification bends of the bentonite chemical structure, except for the observable deviation in the positions of the bands, as well as the strong intensification in the absorption bands of structural hydroxyl groups and interlayered water molecules (Figure 4 (B)). This finding reflects the significant hydration effects of the acid treatment processes and demonstrates the non-extensive destruction effect of the acid activation reactions of the structural units of montmorillonite. 

Regarding the FT–IR spectrum of curcumin as separated components, the observable bands identified the hydroxyl groups of the phenolic phytochemicals (3506 cm^−1^), aromatic C-H of neat curcumin (2943 and 2965 cm^−1^), stretching vibration of C=C (1626.3 cm^−1^), aromatic benzene rings of curcumin (1604.2 cm^−1^), the vibration of C=O (1507 cm^−1^), olefin C-H (1432 cm^−1^), aromatic C-O (1278.6 cm^−1^), aromatic C-H (1162 and 812 cm^−1^), C-O-C (1023 cm^−1^), benzoatetrans-CH (963 cm^−1^), and aromatic cis-CH (716 cm^−1^) (Figure 4 (C)) [30,37,47]. Upon comparing the spectrum of the CU/BE hybridized product with both separated bentonite and curcumin spectra, the resultant spectrum demonstrated complex related organic/inorganic chemical groups but with considerable deviation in their identification absorption bands (Figure 4 (D)). This signified the successful and effective intercalation of bentonite sheets with the phytochemical compounds extracted from curcumin. While Si-O (1008 cm^−1^), Al-O (934 cm^−1^), Si–O–Al (534 cm^−1^), and Si-O-Si (473.2 cm^−1^) were detected clearly as characteristic groups of bentonite, the curcumin phytochemicals were identified significantly by benzene rings (1612 cm^−1^), C=O (1509 cm^−1^), olefin C-H (1440 cm^−1^), aromatic C-O (1284 cm^−1^), and benzoatetrans-CH (921 cm^−1^) (Figure 4 (D)). The same complex organic/inorganic functional groups were also identified in the FT–IR spectrum of ZnO@CU/BE but at shifted positions because of the interaction effects of the decorated ZnO nanoparticles (Figure 4 (E)). Moreover, the stretching vibration of the Zn-O bond was clearly detected by the notable absorption band at 620 cm^−1^ [28,29].

**Figure 5:**
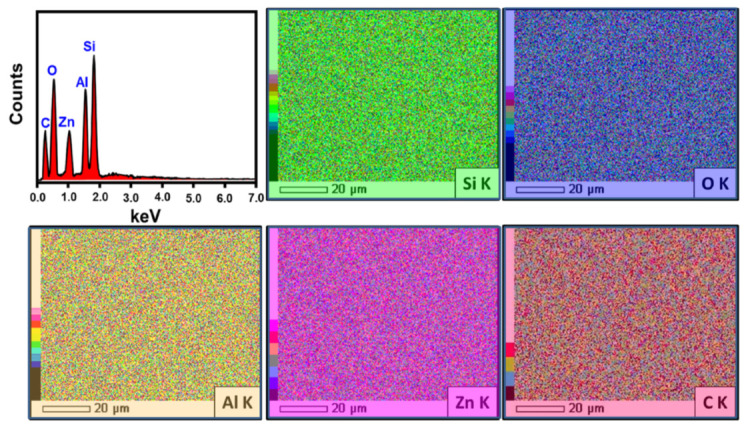
EDX spectrum and mapping of the synthetic ZnO@CU/BE composite.

The previously reported FT–IR findings on the intercalation of bentonite sheets with curcumin-based phytochemicals, as well as the successful decoration of the structure with ZnO, are in agreement with the elemental EDX analysis (Figure 5). The EDX spectrum of the composite (ZnO@CU/BE) demonstrated the presence of the characteristic elements of bentonite (Si and Al), curcumin extract (C), and ZnO (Zn), in addition to the significant oxygen content, which reflected the impact of the intercalated organic phytochemical extracts of curcumin (Figure 5).

### 3.2. Antioxidant Properties

#### 3.2.1. Nitric Oxide Scavenging

**Figure 6:**
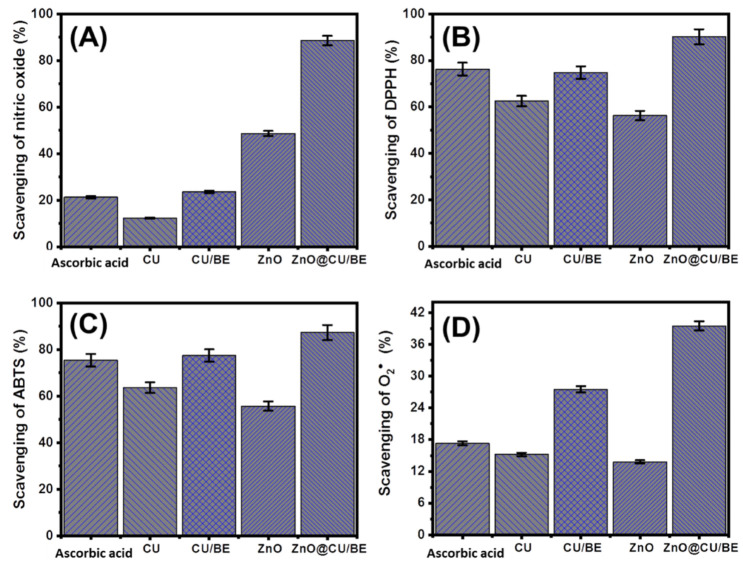
The antioxidant activities of CU, CU/BE, green ZnO, and ZnO@CU/BE structures (100 μg/mL) as compared to ascorbic acid as positive control; (**A**) nitric oxide scavenging activities (20 μL; 100 μg/mL); (**B**) DPPH scavenging activities (20 μL; 100 μg/mL); (**C**) ABTS scavenging activities (10 μL; 100 μg/mL); and (**D**) super oxide radical scavenging activities (100 μL; 100 μg/mL).

Typically, common reactive oxygen species (ROS) are by-products of aerobic respiration and electron transportation. The release of these active radicals results in considerable oxidative stress, which is represented by a controlled increase in intracellular ROS levels and causes several degenerative diseases [51]. Moreover, the presence of nitric oxide free radicals at higher levels may induce damage to cells along with cytotoxic effects on cells, neuronal cell death, and DNA fragmentation [52]. Metals, as well as metal oxide-based materials, especially in their nanoforms, exhibit promising antioxidant properties and are used at effective rates against nitric oxide radicals. The nitric oxide active radicals are gaseous free radicals that exhibit damaging and beneficial biological effects [7,53]. The scavenging and trapping efficiencies of nitric oxide radicals by CU, CU/BE, green ZnO, and ZnO@CU/BE demonstrated the significant potential of the ZnO@CU/BE composite (88.6 ± 1.58%) as compared to that of CU (12.3 ± 1.06%), CU/BE (23.6 ± 1.17%), and green ZnO (48.7 ± 1.36%) as well as the ascorbic acid standard (21.6 ± 1.33%) (Figure 6A).These results validate the preference for applying the composite as a recommended drug candidate compared to the curcumin extract or ZnO, as it demonstrated the best nitric oxide scavenging capacity in addition to the significant biocompatible properties of bentonite. This remarkable enhancement effect of the bentonite host as the carrier might be attributed to the excellent adsorption and absorption capacities of bentonite, leading to the induction of the capture and trapping of nitric oxide. Moreover, it enhanced the stability, dispersion, and exposure of the interactive interfaces between the loaded bioactive compounds (ZnO and curcumin-based phytochemicals) and nitric oxide radicals [24,54]. Finally, the developed composite introduced novel structures of multifunctional active groups with antioxidant affinities towards free nitric oxide active radicals. Moreover, the encapsulation of curcumin extract within the bentonite layers enhanced its antioxidant activity by promoting its solubility compared to the limited solubility of pure curcumin [55].

#### 3.2.2. DPPH Radical Scavenging

The DPPH radical scavenging properties of CU, CU/BE, green ZnO, and ZnO@CU/BE were studied using synergetic procedures. The determined results demonstrated the significant scavenging efficiency of DPPH radicals by the synthetic ZnO@CU/BE composite (90.2 ± 1.76%) compared to that of CU (62.5 ± 1.62%), CU/BE (74.8 ± 1.41%), and green ZnO (56.3 ± 1.37%) as well as the ascorbic acid standard (76.3 ± 1.28%) (Figure 6B). Moreover, the obtained activity by the composite is significantly higher than several investigated structures in the literature such as Ag doped ZnO (73.79%) [7], CuO (about 80%) [56], Ag nanoparticles (67%) [57], RGO-ZnO NCs (69%) [15], Pt nanoparticles (70%) [58], and Au nanoparticles (70.73%) [59]. Generally, the DPPH scavenging mechanism of the prepared ZnO@CU/BE composite involves the significant transfer of an electron (e^−^) and proton (H^+^) to the organic DPPH radical, suggesting coupled proton/electron transfer reactions [5]. The essential antioxidant mechanism is mediated by the loaded ZnO and involves the transmission of the electron density, which is positioned at oxygen, to the common odd electron that is situated at the nitrogen atom within the structure of DPPH [41]. The bentonite carrier exhibited strong inducing effects on a previously reported mechanism. The negatively charged surface of the bentonite layers might enhance the charge separation efficiency via electron attraction processes, which, in turn, enhances the antioxidation activity of the final composite (ZnO@CU/BE) [60].

#### 3.2.3. ABTS Radical Scavenging

The ABTS scavenging test has recently been recommended to evaluate the antioxidant properties of different compounds, especially complex structures and hybrid materials. This assay involves the generation of ABTS cation radical (ABTS^●+^) using potassium persulfate. Therefore, the synthetic structures that exhibit hydrogen-donating antioxidant properties, as detected in the synthetic ZnO@CU/BE (ZnO and curcumin), will be very effective in reducing the released ABTS^●+^. The scavenging properties of CU, CU/BE, green ZnO, and ZnO@CU/BE against the ABTS radical were studied using synergetic procedures, with ascorbic acid as a control (Figure 6C). The determined results are in agreement with previously reported scavenging results for other radicals. These results reflected the higher activity of the synthetic ZnO@CU/BE composite (87.3 ± 1.61%) than that of CU (63.7 ± 1.35%), CU/BE (77.4 ± 1.83%), green ZnO (55.7 ± 1.44%), and ascorbic acid standard (75.4 ± 1.14%) (Figure 6C). The recognized ABTS scavenging activity by the composite is higher than the reported activity using several investigated materials such as oregano oil/halloysite [61], mung bean protein [62], soy β-conglycinin-dextran-polyphenol [55], and chitosan/lignosulfonate micelles [63]. In addition to the previously reported enhancement effects of the bentonite carrier on the physicochemical properties of the loaded green ZnO, the wrapping of curcumin-based phytochemicals between the bentonite layers effectively contributed to the generation of hydrogen atoms [24]. 

#### 3.2.4. Superoxide Radical Scavenging

Active superoxide anion radicals (O_2_^●−^) are produced mainly by cellular organelles, such as mitochondria, and are immediately transformed into active hydroxyl radicals (^•^OH) and hydrogen peroxide (H_2_O_2_). The human body exhibits a natural defense system against superoxide radicals and other reactive radicals to maintain effective physiological homeostasis; however, sometimes nonfunctional or noneffective values are also observed, as has been widely detected in various diseases. The uncontrolled release of active oxygenated radicals and the nonregulation of their levels are associated with severe pathophysiological conditions and, in turn, degenerative diseases that pose significant health risks [64]. The determined results demonstrate strong scavenging percentages of O_2_^●−^ associated with the synthetic ZnO@CU/BE composite (39.5 ± 1.12%) as compared to those related to CU (15.2 ± 1.66%), CU/BE (27.5 ± 1.31%), and green ZnO (13.8 ± 1.22%) as well as the ascorbic acid standard (17.3 ± 1.34%) (Figure 6D). Moreover, the obtained activity by the composite is significantly higher than several investigated structures in the literature such as Ag doped ZnO (27.34%) [7], CuO (about 80%) [56], and Ag nanoparticles [57]. Therefore, the prepared ZnO@CU/BE green structures can be deemed promising scavenging or trapping agents for superoxide radicals. This provides protection against cellular damage as it can strongly hinder the interaction between superoxide species and cellular molecules, including RNA, DNA, and proteins [65]. 

### 3.3. Antidiabetic Properties

#### 3.3.1. Porcine Pancreatic α-Amylase Inhibition Assay

**Figure 7:**
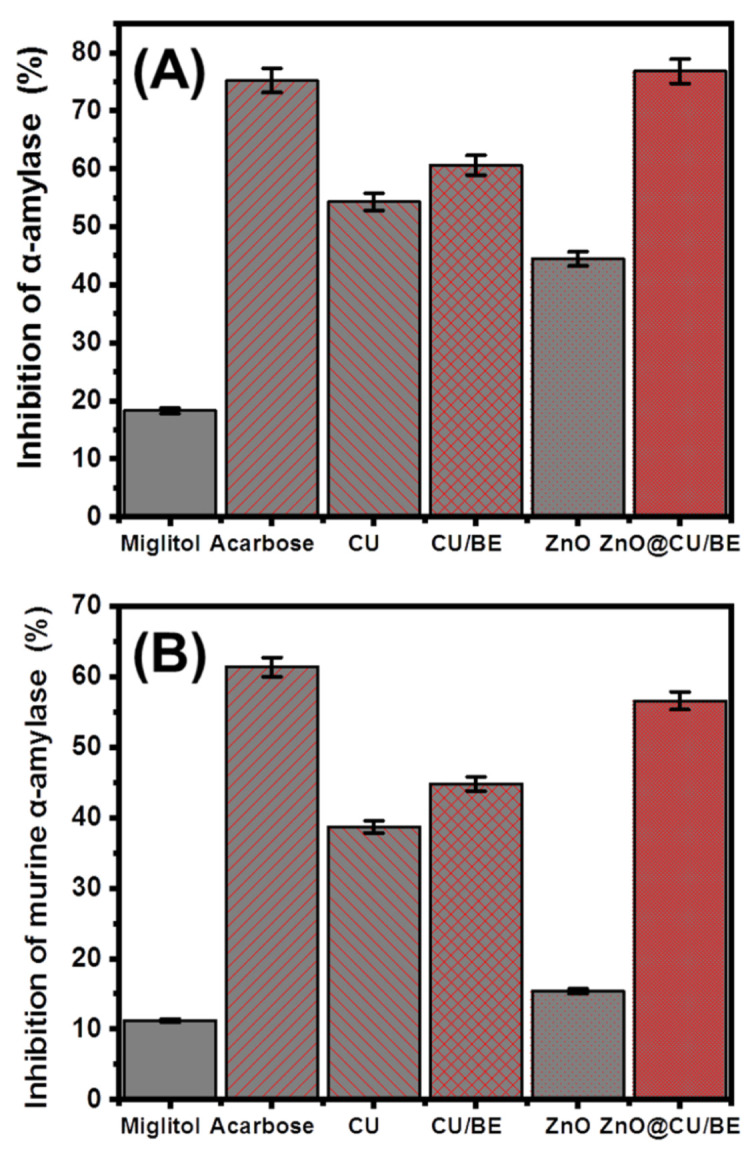
The α-amylase inhibition activities of CU, CU/BE, green ZnO, and ZnO@CU/BE structures as compared to the positive control miglitol and acarbose (500 μL; 100 μg/mL); (**A**) porcine pancreatic α-amylase enzyme (100 μL; 50 μg/mL); and (**B**) murine pancreatic α-amylase enzyme (100 μL; 50 μg/mL).

The inhibitory effects of the commonly used antidiabetic structures on α-amylase were examined. The results exhibited significant preventive effects on the breakdown reactions of complex sugars into simple chemicals, which strongly hinders the absorption efficiency of dietary starches and, in turn, controls postprandial hyperglycemia in diabetes [28]. The α-amylase inhibitory activities of CU, CU/BE, green ZnO, and ZnO@CU/BE were determined in synergetic studies and are presented in Figure 6A. The inhibitory properties of the CU extract, CU/BE, green ZnO, and ZnO@CU/BE prepared structures against porcine pancreatic α-amylase were significantly enhanced at different stages of the hybridization process (Figure 7A). The determined inhibition percentages of CU extract and CU/BE were 54.3 ± 1.46% and 60.7 ± 1.77%, respectively (Figure 7A). An enhancement of approximately 6.4% was observed after the intercalation of curcumin extracts within the layered structure of montmorillonite, which might be attributed to the role of the montmorillonite host in controlling the release rate of loaded active phytochemicals and, in turn, preserving their impact on the enzyme for long intervals. The determined inhibition percentage of green ZnO was 44.5 ± 1.53% which was significantly lower than the determined value of ZnO@CU/BE (76.8 ± 1.87%). This value was higher than the values of CU extract and CU/BE as well as those of the assessed standards of the commercially used drugs (miglitol (18.3 ± 1.42%) and acarbose (75.2 ± 1.68%) (Figure 7A). This might be attributed to the significantly high surface area of the bentonite layers, which enhanced the dispersion, stability, and exposure properties of the supported green ZnO nanoparticles and prevented adverse agglomeration, thereby, increasing the interaction area or interface with the enzyme. The aggregation and agglomeration of synthetic nanoparticles lead to the degeneration of their biological activities [24]. This is in agreement with the results of previous studies examining the impact of carriers on enhancing the antidiabetic and biological activities of curcumin and ZnO [15,24,30,66]. The recognized activity of ZnO@CU/BE is significantly higher than several studied structures in the literature such as Ag doped ZnO (23%) [7], Ag/CuO (38.9%) [67], RGO-ZnO (51.19%) [15], Ag nanoparticle (61%) [57], NiO nanoparticles (22%) [67], and synthetic Fe-CuO-SiO_2_ composite [68].

The α-amylases are effective and dominant digestive enzymes that exhibit a strong capacity to break down the long-chain polymeric structure of carbohydrates, such as polysaccharide starch into smaller structures of maltose, which are immediately transformed into glucose forms [7]. Therefore, their effective inhibition significantly reduces blood sugar levels [69]. The results obtained using CU/BE, green ZnO, and ZnO@CU/BE validate their significant potential for use as antidiabetic agents, considering the reported value of the commonly used drugs miglitol and acarbose. Some of the commercially used enzyme inhibitors, such as acarbose, exhibit significantly high inhibition values at the highest concentrations. However, they are still expensive compared to natural products, in addition to the commonly demonstrated side effects with their applications [70,71].

#### 3.3.2. Murine Pancreatic α-Amylase Inhibition

The inhibitory potential of CU extract, CU/BE, green ZnO, and ZnO@CU/BE structures was evaluated against crude murine pancreatic α-amylase. However, the recognized results demonstrate lower inhibition effects than the reported results against porcine pancreatic α-amylase; the results signify the enhancement in the inhibition activity with the different hybridization processes. The application of CU extract and CU/BE resulted in inhibition percentages of 38.7 ± 1.33% and 44.8 ± 1.52%, respectively, with notable enhancement by approximately 6% (Figure 7B). The application of ZnO@CU/BE showed an inhibition percentage of 56.5 ± 1.67%, which was higher than the determined value of green ZnO (15.4 ± 1.11%) by approximately 41%, compared to that of CU which was 17.8% and that of CU/BE which was 11.7% (Figure 7B). These results validate the significant inhibition properties of the synthetic ZnO@CU/BE composite against both the tested commercially available enzymes and the metabolically crude active enzymes considering the determined results using the controls miglitol (11.2 ± 1.61%) and acarbose (61.4 ± 1.55%) (Figure 7B).

#### 3.3.3. Pancreatic α-Glucosidase Inhibition

**Figure 8:**
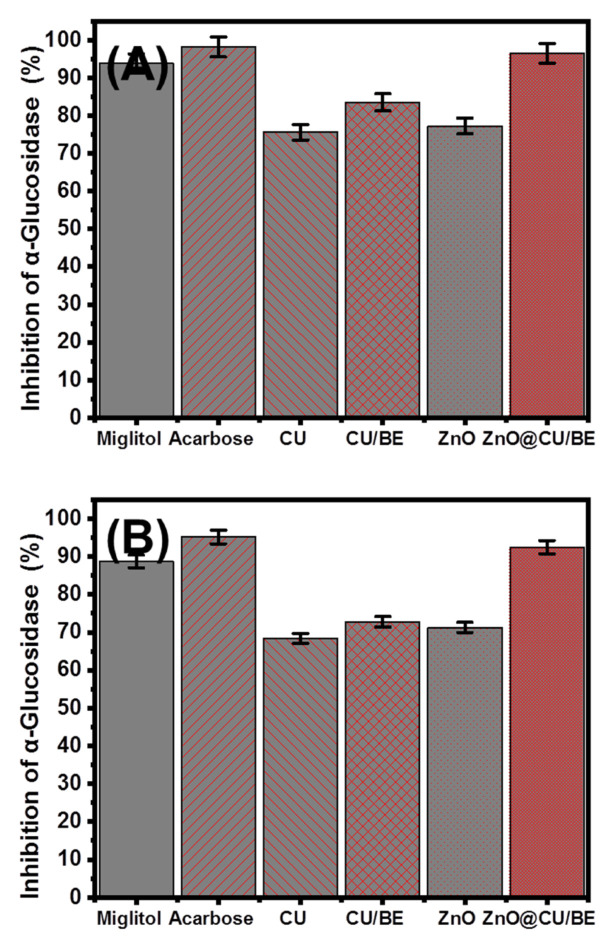
The α-glucosidase inhibition activities of CU, CU/BE, green ZnO, and ZnO@CU/BE structures as compared to the positive control miglitol and acarbose (200 μL; 100 μg/mL); (**A**) pancreatic α-glucosidase enzyme (100 μL; 0.1 unit/mL); and (**B**) murine intestinal α-glucosidase enzyme (100 μL; 0.1 unit/mL).

Pancreatic and intestinal glycosidases are the most effective enzymes for the metabolism of dietary carbohydrates. Therefore, their inhibition demonstrates strong retarding effects on the absorption of glucose and, in turn, induces the suppression of hyperglycemia. The inhibitory properties of CU extract, CU/BE, green ZnO, and ZnO@CU/BE against α-glucosidase were determined and compared with those of commercially used miglitol and acarbose drugs, which were used as controls (Figure 8A). The determined percentages of inhibition against the assessed α-glucosidase enzyme corresponding to CU extract, CU/BE, green ZnO, and ZnO@CU/BE were 75.6 ± 1.37%, 83.5 ± 1.48%, 77.3 ± 1.27%, and 96.5 ± 1.07%, respectively (Figure 8A). This remarkable enhancement in the inhibition properties indicated the significant effect of the bentonite carrier in ensuring the stability, dispersal properties, exposure, and interactive interface of the effectively loaded functional groups of ZnO, as well as the extracted phytochemical curcumin. Moreover, the green fabrication of ZnO using the curcumin extract itself might result in the formation of curcumin/ZnO complexes or ZnO-capped curcumin structures that might exhibit potent inhibition properties, based on the previous literature [17]. The recognized activity of ZnO@CU/BE against α-glucosidase is significantly higher than several studied structures in the literature such as Ag doped ZnO (91.19%) [7], Ag/CuO (19.6%), and RGO-ZnO (53.24%) [15].

#### 3.3.4. Murine Intestinal α-Glucosidase Inhibition

The synthetic products were also assessed as potential inhibitors of murine intestinal glucosidase. The synthetic hybridized products (CU/BE and ZnO@CU/BE) showed notable enhancement in the percentage inhibition against the evaluated murine intestinal glucosidase enzyme compared to the separated phases of curcumin extract and the green-fabricated ZnO based on the phytochemicals of the curcumin extract. The determined percentages of inhibition of the CU extract, CU/BE, green ZnO, and ZnO@CU/BE were 68.4 ± 1.27%, 72.8 ± 1.51%, 71.3 ± 1.16%, and 92.5 ± 1.10%, respectively (Figure 8B). Previous studies have reported a reduction in blood glucose levels using ZnO-based materials as antidiabetic agents, in addition to its effect of increasing the serum insulin, insulin receptor, glucokinase activity, and glucokinase genes [72]. Therefore, the present study demonstrated the development of a highly effective multifunctional antidiabetic structure (ZnO@CU/BE) at a low fabrication cost using simple and green methods based on natural resources and plant extracts. 

#### 3.3.5. Amyloglucosidase Inhibition

**Figure 9:**
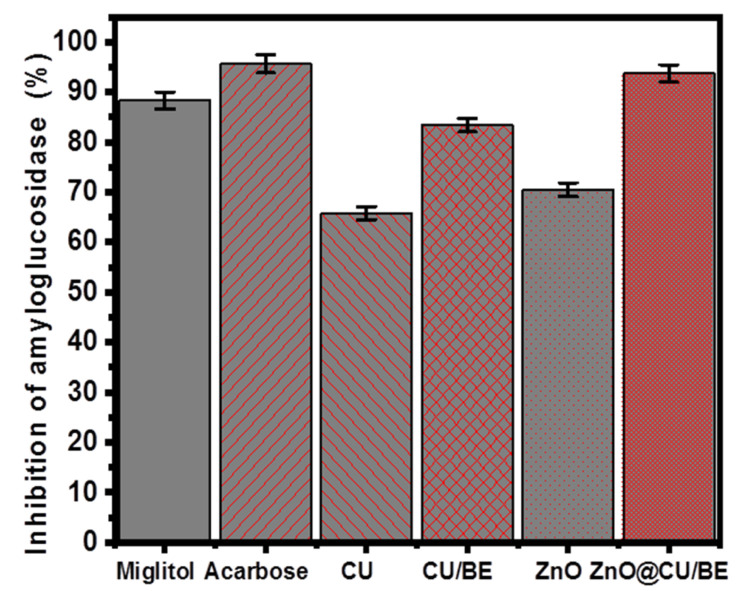
The amyloglucosidase (100 μL; 0.1 unit/mL) inhibition activities of CU, CU/BE, green ZnO, and ZnO@CU/BE structures as compared to the positive control miglitol and acarbose (100 μL; 100 μg/mL).

The properties of the synthetic ZnO@CU/BE structure as an antidiabetic agent in comparison with its integrated components were also based on their inhibitory effects on amyloglucosidase. The reported results also demonstrated significant inhibition of the enzyme, with a remarkable augmentation in the inhibitory effects in the presence of the hybridized products (CU/BE and ZnO@CU/BE) rather than the separated phases of curcumin extract and the synthetic green ZnO nanoparticles (Figure 9). The observed percentages of inhibition of CU extract, CU/BE, green ZnO, and ZnO@CU/BE against amyloglucosidase enzyme were 65.8 ± 1.35%, 83.4 ± 1.47%, 70.5 ± 1.77%, and 93.7 ± 1.55%, respectively which are promising values considering the estimated inhibition percentages of the commercial drug used as controls (miglitol (88.3 ± 1.83%) and acarbose (95.6 ± 1.72%)) (Figure 9). Considering the previous results in comparison with the drug standards, the synthetic ZnO@CU/BE green composite can be used as a starch blocker with significant efficiency and can be applied to prevent the absorption of dietary starches by the human body owing to its inhibitory effects on the conversion of the complex sugar species into simple forms [72].

## 4. Conclusions

A green ZnO-decorated acid-activated bentonite-mediated curcumin extract (ZnO@CU/BE) composite was synthesized via a facile green method using curcumin extract as an enhanced antioxidant and antidiabetic agent with multifunctional properties (curcumin-based phytochemicals, zinc oxide-capped curcumin, and zinc/curcumin complexes). It shows enhanced antioxidant and antidiabetic activities against the commonly studied radicals [nitric oxide (88.6 ± 1.58%), DPPH (90.2 ± 1.76%), ABTS (87.3 ± 1.61%), superoxide (39.5 ± 1.12%)] and enzymes [porcine pancreatic α-amylase (76.8 ± 1.87%), murine pancreatic α-amylase (56.5 ± 1.67%), pancreatic α-Glucosidase (96.5 ± 1.07%), murine intestinal α-Glucosidase (92.5 ± 1.10%), and amyloglucosidase (93.7 ± 1.55%)] compared to the commercial drugs and the separated components of the ZnO@CU/BE composite. The findings reflect the enhancement effect of bentonite carriers on the biological activities of curcumin-based phytochemicals and green ZnO. Therefore, the synthetic green ZnO@CU/BE composite can be recommended as enhanced, low-cost, biocompatible, safe, and simply produced antioxidant and antidiabetic agents compared to the commercially used drugs which will be covered by future in vivo studies.

## Abbreviation

ZnOZinc oxideCUCurcuminBEBentoniteCU/BEcurcumin intercalated bentoniteZnO@CU/BEZnO-decorated acid-activated bentonite-mediated curcumin extractDPPH1, 1-diphenyl-2-picrylhydrazil PNPGpara-nitrophenyl α-glucopyranoside (PNPG)ABTS2,2′-azino-bis(3-ethylbenzothiazoline-6-sulphonic acidDNSA5-dinitrosalicylic acidO_2_^●−^superoxide anion radical^•^OHhydroxyl radicalROSreactive oxygen speciesLOILoss of ignition ANOVAanalysis results of varianceXRDX-ray diffraction patternSEMScanning electron microscope HRTEMTransmission electron microscopeFT-IRFourier-transform infrared

## Data Availability

Data are available upon reasonable, by the Corresponding Authors.
